# Child mortality in Bangladesh – why, when, where and how? A national survey-based analysis

**DOI:** 10.7189/jogh.11.04052

**Published:** 2021-09-11

**Authors:** Ahmed Ehsanur Rahman, Aniqa Tasnim Hossain, Abu Bakkar Siddique, Sabrina Jabeen, Mohammod Jobayer Chisti, David H Dockrell, Harish Nair, Kanta Jamil, Harry Campbell, Shams El Arifeen

**Affiliations:** 1 International Centre for Diarrhoeal Disease Research, Bangladesh; 2 Usher Institute, University of Edinburgh, Edinburgh, UK; 3 USAID, Washington DC, USA

## Abstract

**Background:**

Updated information on the cause of childhood mortality is essential for developing policies and designing programmes targeting the major burden of disease. There is a paucity of evidence regarding the current estimates of the cause of death in Bangladesh, which is essential for reinvigorating the current policies and reshaping existing strategies to avert preventable deaths. This paper aims to address this critical evidence gap and report the cause, timing and place of death among children under-five years of age using a nationally representative sample.

**Methods:**

The present study was undertaken to provide updated estimates of causes of death among children under-five years of age using data from the 2017-18 round of the Bangladesh Demographic and Health Survey (BDHS). The verbal autopsy (VA) questionnaire of the 2017-18 BDHS was adapted from the standardised WHO 2016 instruments. Specially trained physicians reviewed the responses of the VA questionnaire and assigned the cause of death based on the online-2016-version of the International Classification of Diseases (ICD-10). We included 456 deaths among children under-five years of age in our analysis. Descriptive statistics were used to present the causes, timing and places of death with uncertainty ranges (UR).

**Results:**

Pneumonia is the major killer (19%), accounting for approximately 24 268 (UR = 21 626-26 695) under-five deaths per-year. It is followed by birth asphyxia (16%), prematurity and low-birth-weight (11%), serious infections including sepsis (8%) causing 20 882 (UR = 18 608-22 970), 14 956 (UR = 13 327-16,452), and 10 723 (UR = 9555-11,795) deaths per-year, respectively. Drowning (8%) caused 10 441 (UR = 9304-11 485) deaths and congenital anomaly (7%) resulted in d 8748 (UR = 7795-9623) deaths per-year. Around 29% of all deaths occurred on the first day, 52% within the first week, and 66% within the first month of life. Around 70% of birth asphyxia, prematurity, and low birth weight-related deaths happen on the day of birth. Approximately 43% of pneumonia-related deaths occur in age 1-11 months, and around 51% of drowning-related deaths happen in age 12-23 months.

**Conclusions:**

Pneumonia with other serious infections, birth asphyxia, prematurity and low-birth-weight are responsible for more than half of all deaths among children under-five years of age. Strengthening the existing maternal, neonatal and child health programmes may be helpful in averting the majority of these preventable deaths. A multisectoral approach is required for the prevention of childhood deaths, especially drowning-related fatalities. Special measures need to be taken to prevent and control emerging public health challenges like birth defects and congenital anomalies.

The rapid decline in childhood mortality observed in Bangladesh during the first two decades since the 1990s came to an apparent stalling in the 2010s [1,2]. Today, far too many of these children die due to preventable causes. According to the Bangladesh Demographic and Health Survey, the neonatal mortality rate was 28 per thousand live births in 2014 and 30 per thousand live births in 2017 [1,2]. Similarly, the under-five mortality rate was 46 per thousand live births in 2014 and 45 per thousand live births in 2017 [1,2]. The Government of Bangladesh (GoB) needs to critically review the existing policies, strategies and programmes to identify possible gaps related to newborn and child health and identify strategies to avert preventable deaths.

Prioritising interventions targeting the major causes of death can provide fresh impetus for accelerating the reduction in mortality rates. In high-income settings, the civil registration system is the most common and valid source of data as it systematically documents vital statistics related to deaths and their causes [3]. Unfortunately, there are substantial gaps in coverage and quality of birth and death reporting in low- and middle-income settings [3]. In Bangladesh, the coverage of birth registration is around 54%, and death registration is approximately 13% [4,5]. Moreover, about 70% of deaths take place outside health facilities with minimum to no information regarding the cause of death [6]. Out of the facility deaths, very few have medical certification of the cause of death, as the programme is in its early roll-out phase [6]. The Bangladesh Bureau of Statistics runs surveillance with a nationally representative sample to track key demographic indicators. However, information regarding the cause of death is not systematically collected by adopting a globally accepted standard [7]. icddr,b, an international health research organisation based in Bangladesh, manages several demographic surveillance sites throughout Bangladesh and collects information on the cause of death using the WHO recommended verbal autopsy tools [8]. Although these demographic surveillance sites offer a rich source of data on the cause of death patterns and trends over time, the estimates are not nationally representative. In this regard, the last national estimate was based on the verbal autopsy conducted among under-five deaths in the 2011 round of the Bangladesh Demographic and Health Survey (BDHS) [9]. Although another round of BDHS was conducted in 2014, it did not include the verbal autopsy component. Therefore, there is a paucity of evidence regarding the current national estimates of the cause of death, which is essential for reinvigorating the current policies and reshaping existing strategies to avert child deaths.

This paper aims to address this critical evidence gap and report the timing, place, and cause of death among children under-five years of age with a nationally representative sample.

## METHODS

We analysed publicly available data from the 2017-18 round of BDHS. BDHS uses a nationally representative sample, and the 2017-18 round collected information on the cause of death through verbal autopsy. The verbal autopsy tool was adapted from the standardised and validated WHO 2016 and 2011 BDHS verbal autopsy instruments [2]. The tool was adapted to Bangladesh's context based on expert review and translated to Bangla, the most commonly spoken language in Bangladesh. The 2017-18 tool had a separate questionnaire for neonatal deaths (age 0-28 days) and child deaths (age 29 days to 59 months). The questionnaires included both open-ended and close-ended questions and were pretested in two rural clusters in Manikgonj district and two urban clusters in Dhaka before finalisation. A national advisory committee constituted by verbal autopsy experts supervised the country adaptation, translation and pretesting processes for finalisation.

Mitra and Associates collected the data under the leadership of National Institute of Population Research and Training (NIPORT) [2]. The verbal autopsy data collection team had trained interviewers and field editors. The data collection team, the supervisors and the quality controllers received one-week training from icddr,b on the VA questionnaire before data collection. During the survey, four review meetings were organised to discuss the quality of data collection and address specific problems faced by interviewers. The VA data collection team conducted in-person interviews with the next of kin or the primary caregivers of all children under-five years of age who died within five years of the survey in the surveyed areas. All interviews were successfully completed except one where the respondent refused to participate.

icddr,b was responsible for assigning the cause of death through physician review. Three physicians, with a degree of Bachelor in Medicine and Surgery (MBBS), were recruited by icddr,b and received a training for four consecutive weeks on the verbal autopsy instruments and cause of death assignment based on the online-2016-version of the International Classification of Diseases (ICD-10) [10]. The physicians were trained by master trainers who were previously involved with 2011 BDHS, 2013 Bangladesh Urban Health Survey and 2016 Bangladesh Maternal Mortality Survey.

Each questionnaire was independently reviewed by two physicians and each physician assigned a cause of death (direct, underlying and contributory). When the two physicians agreed on the underlying cause, it was considered the final cause of death. In case of disagreement, an additional review was conducted by a third physician. If the underlying causes were agreed upon by any two of three physicians, it was considered the final cause of death. The cause of death was recorded as “undetermined” if no agreement was reached after the third physician review. In a few cases, where two physicians had assigned identical sets of causes of deaths but disagreed on whether these were immediate or underlying causes, a discussion was arranged to reconcile the differences in the presence of a trained verbal autopsy expert. The review process is summarised in Figure S1 in the **Online Supplementary Document****.**

### Analysis plan

We used Stata version 14 for data analysis [11]. We used descriptive statistics to report the cause, timing and place of death. We grouped similar ICD-10 codes and presented the cause of death in 16 broad categories. Table S1 in the **Online Supplementary Document** shows the ICD-10 codes that were grouped under each broad category of cause of death. We reported the proportional distribution of the cause of death among all under-five deaths, neonates (0-28 days) and post-neonatal children (29 days-59 months). We used the under-five mortality rates and the cause-specific mortality fractions to estimate the cause-specific mortality rates. The uncertainty range (UR) was calculated using the confidence intervals obtained for the reported mortality rate among children under-five years of age in 2017-18 BDHS. The cause-specific mortality rates were also disaggregated by sex, birth order, father and mother’s education, residence (rural-urban), and wealth (lowest and highest quintile). The sample size was not enough to check for statistically significant difference in the cause specific mortality rates by disaggregating variables. Hence, we considered a difference ‘substantial’ if the difference was ≥25% (relative) by disaggregating variables. We also projected the number of deaths for each of the broad categories using the adjusted population size in 2015 [12], crude birth rate from the 2015 Sample Vital Registration System report [13] and the cause-specific mortality rates reported in our paper. The detailed calculations are presented in Table S2 in the **Online Supplementary Document**.

The overall timing of death was presented by age in months. Also, we showed the timing of death in days for neonatal (0-28 days) mortality. Place of death was categorised as home, facility (public or private) and during transportation. We also presented the timing of death and place of death disaggregated by the cause of death. The time variable has been further disaggregated to present the cause of death by the following categories: Day 0, Day 1-6, Day 7-28, 1-11 months, 12-23 months, 24-35 months, 36-47 months and 48-59 months.

All analysis presented in this paper were weighted for the overall sampling frame of 2017-18 BDHS.

### Ethical approval

This study used secondary data set collected by National Institute of Population Research and Training (NIPORT), Bangladesh, and Monitoring and Evaluation to Assess and Use Results Demographic and Health Surveys (MEASURE DHS). We got approval from the DHS program for utilising the applicable data sets for this study.

## RESULTS

We included all 456 deaths among children under-five years of age in our analysis.

**Figure 1:**
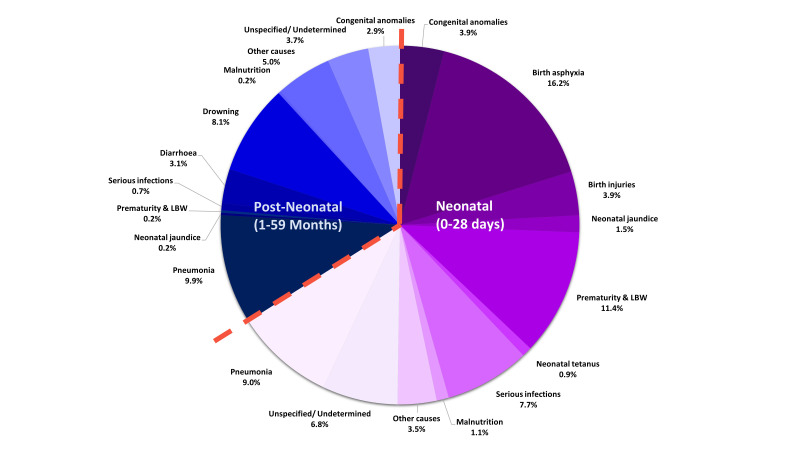
Distribution of cause of death among children under-five years of age, presented in percent distribution, N = 456.

**Figure 1** presents the percent distribution of the cause of death among all children under-five years of age. Pneumonia is the major killer (9% during the neonatal and 10% during the post-neonatal periods), followed by birth asphyxia (16% exclusively during the neonatal period), prematurity and low-birth-weight (11% during the neonatal and one during the post-neonatal periods), serious infections including sepsis (8% during the neonatal period and 1% during the post-neonatal period), drowning (8% exclusively during the post-neonatal period), congenital anomaly (4% during the neonatal and 3% during the post-neonatal periods) and birth injuries (4% during the neonatal period). Although less than 1% of deaths were directly attributable to malnutrition, it was associated with 13% of all under-five deaths (not presented in the figure).

**Figure 2:**
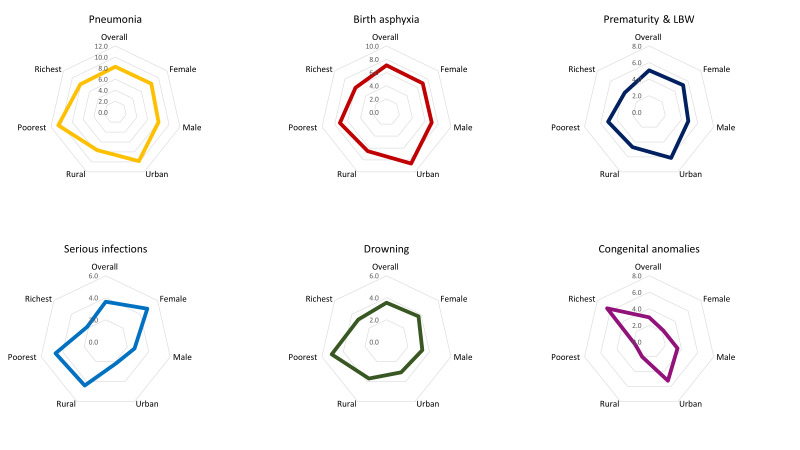
Cause specific mortality by background characteristics, presented in deaths per thousand live births.

**Figure 2** presents the cause-specific mortality rates by background characteristics. The cause-specific mortality rate was 8.5 per thousand live births for pneumonia (UR = 7.6-9.3), 7.3 per thousand live births for birth asphyxia (UR = 6.5-8.0), 5.5 per thousand live births for prematurity and low-birth-weight (UR = 4.7-5.8), 3.8 per thousand live births for serious infections (UR = 3.3-4.1), 3.7 per thousand live births for drowning (UR = 3.3-4.0), 3.1 per thousand live births for the congenital anomaly (UR = 2.7-3.4), 1.8 per thousand live births for birth injuries (UR = 1.6-2.0) and 1.4 per thousand live births for diarrhoea (UR = 1.2-1.5). We did not observe any substantial difference by gender across the mortality rates for different causes, except serious infection, where it was higher among female children. While the cause-specific mortality rates for birth asphyxia, congenital anomaly and birth injury were higher among the first-born children, we observed lower rates (in the same group) for pneumonia, drowning and diarrhoea. For most of the categories, the cause-specific mortality rates were higher among both mothers and fathers who did not have any formal education than those with ten or more years of schooling. The cause-specific mortality rates for pneumonia, birth asphyxia, prematurity and low birth weight, congenital anomaly and birth injury were higher in urban areas. However, the cause-specific mortality for serious infection was higher in rural areas. We did not observe any notable difference in the cause-specific mortality rates for drowning and diarrhoea based on the deceased children's residence status. The cause-specific mortality rates for pneumonia, birth asphyxia, prematurity and low birth weight, serious infection, drowning and diarrhoea were higher among the poorest than the richest. On the contrary, the cause-specific mortality rate for the congenital anomaly was higher among the richest than the poorest. Table S3 in the **Online Supplementary Document** presents more disaggregated estimates of cause specific mortality rates. Figure S2 in the **Online Supplementary Document** compares the major causes of death between 2011 BDHS and 2017-18 BDHS by cause-specific mortality rates.

**Figure 3:**
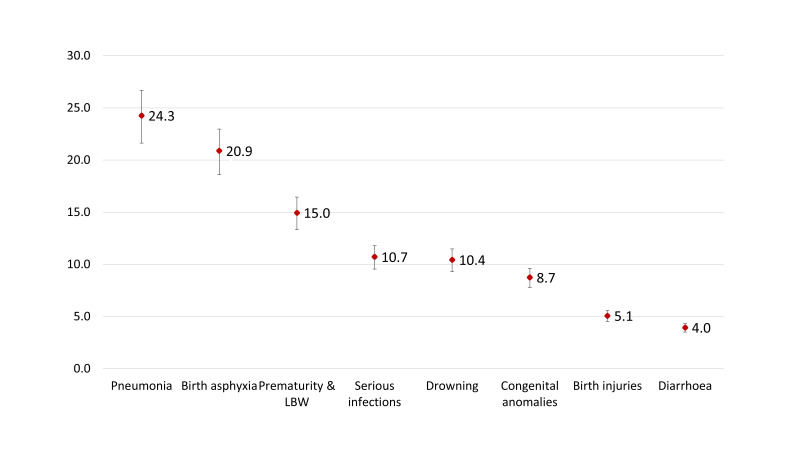
Estimated number of deaths in 2015 by the cause of death, presented in thousand.

**Figure 3** summarises the estimated number of deaths among children under-five years of age in 2015 by causes. Pneumonia caused an estimated 24 268 (UR = 21 626-26 695) deaths, and birth asphyxia caused approximately 20 882 (UR = 18 608-22 970) deaths. Approximately 15 000 (UR = 18 608-22 970) children died of prematurity and low-birth-weight. Serious infections including sepsis caused 10 723 deaths (UR = 9555-11 795) and drowning were responsible for about 10 441 (UR = 9304-11 445) deaths. Birth defects and congenital anomalies were responsible for around 8748 (UR = 7795-9623) deaths. Around 5079 (UR = 4526-5587) deaths were attributable to birth injuries and close to 3951 (UR = 3520-4436) deaths were due to diarrhoea.

**Figure 4:**
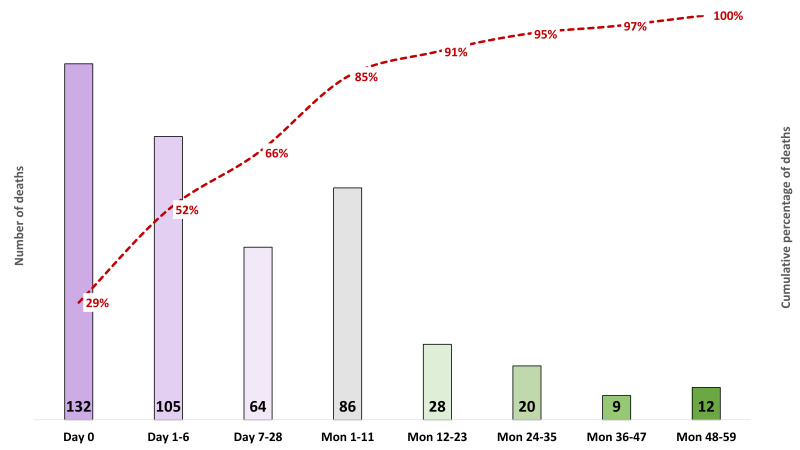
Timing of under-five deaths, presented in numbers and cumulative percentage, N = 456.

**Figure 4** shows the timing of death where the primary vertical axis presents the number of deaths, and the secondary vertical axis presents the cumulative percentage. Around 29% of all deaths occurred on the day of birth, 52% died within the first week, 66% in the first month and 85% within the first year.

**Figure 5:**
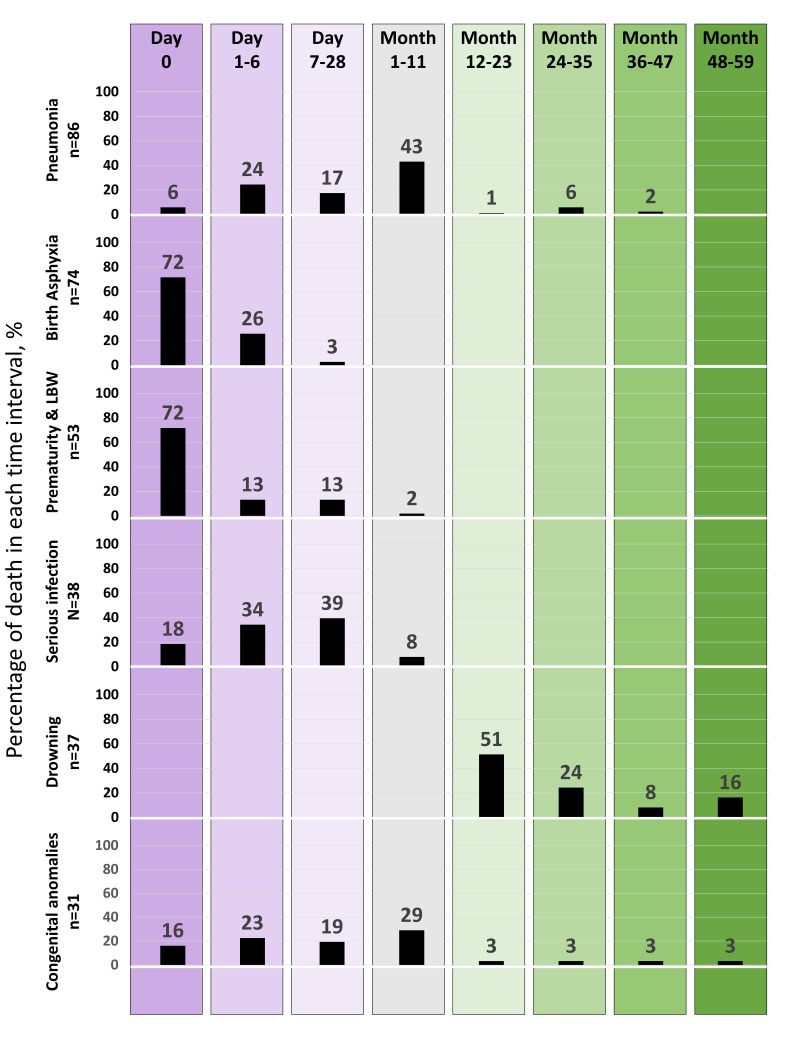
Timing of under-five deaths by causes, presented in percent distribution.

**Figure 5** presents the percent distribution of the timing of under-five deaths by causes. Close to half of the pneumonia-related deaths happened in the first month of life, and 43% between month 1 and month 11. Less than 10% of the pneumonia-related deaths occurred after the infancy period (0-11 months). Among those who died due to birth asphyxia, approximately 70% died on the day of birth, 26% died between day 1 and 6, and the rest died within the first one month. Similarly, 72% of all prematurity and low birth weight-related deaths occurred on the day of birth, and another 13% died between day 1 and day 6. The risk of serious infection-related death was the highest between day 7 and day 28 (39%), followed by day 1 and day 6 (34%) and the day of birth (18%). On the contrary, all deaths due to drowning occurred after the infancy period, particularly between months 12 and 23 (51%). Another 24% of drowning-related deaths happened between month 24 and month 35.

**Figure 6:**
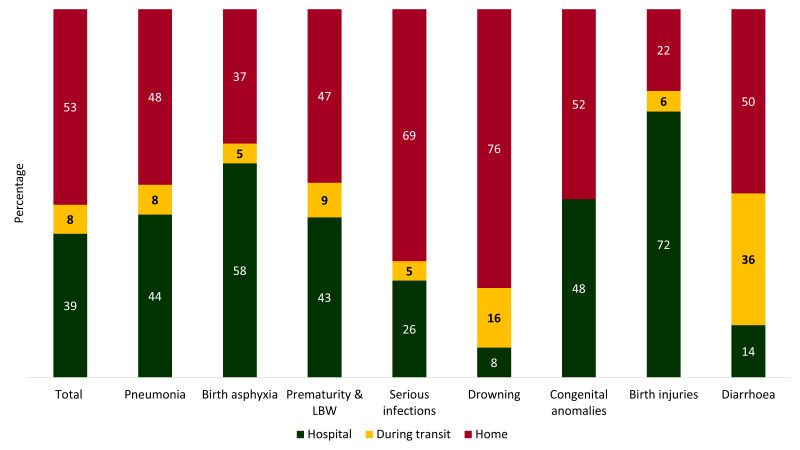
Place of under-five deaths, presented in percent distribution by the cause of death, N = 456.

**Figure 6** presents the percent distribution of place of death by causes. Out of all deaths among children under-five years of age, 53% occurred at home, 39% at hospitals or health facilities and 8% during transit. Among the pneumonia deaths, 48% happened at home, 44% at hospitals and 8% during transit. 58% of deaths due to birth asphyxia and 72% of the deaths due to birth injuries occurred at hospitals. On the contrary, 69% of the deaths due to serious infections and 76% of the deaths due to drowning took place at home. Among the deaths attributable to diarrhoea, 36% happened during transit.

**Figure 7:**
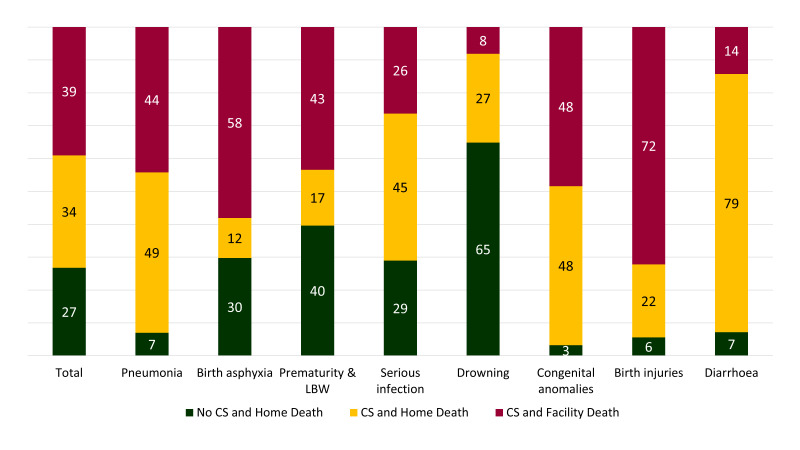
Care seeking (CS) practice before under-five deaths, presented in percent distribution by causes, N = 456.

**Figure 7** summarises the care-seeking practice before death among children under-five years of age. Among the pneumonia deaths, 7% did not seek care, and another 49% died at home after seeking care. Regarding deaths due to prematurity and low birth weight, 40% did not seek care, and 17% died at home after seeking care. Similarly, 7% of the deaths due to diarrhoea did not seek care at all, and another 79% died at home after seeking care from outside the home.

Among the deaths due to pneumonia, around 20% of the parents had doubts about whether medical care was required, 45% received injectable antibiotics, 11% reported problems with hospital admission/medical treatment/getting medication/medical tests and 50% reported that the total cost of care prohibited other household payments.

Among the deaths due to birth asphyxia, around half had facility birth, and 70% had vaginal births. Similarly, 62% of the birth injury-related deaths had facility births, and 85% had vaginal births. Regarding deaths attributable to prematurity and low birth weight, 77% had facility births, and 81% had vaginal births.

Among the deaths due to serious infections, 34% were born premature (as reported by the parents), 13% of the parents had doubts whether medical care was required, 21% received injectable antibiotics, 13% reported problems with hospital admission/medical treatment/getting medication/medical tests and 37% reported that the total cost of care prohibited other household payments.

Regarding deaths due to diarrhoea, 43% of the parents had doubts whether medical care was required and 14% did not receive any oral rehydration saline.

## DISCUSSION

Bangladesh has made remarkable progress in health and development since the 1990s [14]. But the recent phase of apparent stalling in reducing neonatal and child morality essentially exposes the disconnect between the fast-changing landscape of child health and the gaps in prioritisation and programme planning [2,15]. This calls for reshaping the existing policies, redirecting the current strategic focus based on updated evidence, and targeting the most pressing health needs. This paper addresses a key evidence gap related to the cause and context of child mortality in Bangladesh. This can aid in reinvigorating the programmes and make course corrective measures to achieving the ambitious Sustainable Development Goal (SDG) targets of reducing neonatal mortality rate to ≤12 per thousand live births and under-five mortality rate to ≤25 per thousand live births and by 2030 [16].

We found that the cause-specific mortality rate due to pneumonia was 8.5 per thousand live birth in 2017-18, making it the largest killer among children under five years of age in Bangladesh. The current rate is much higher than the global average of 6.6 per thousand live births in 2015. It is also higher than the average rate of 7.4 per thousand live births among developing countries and other neighbouring countries like India and Nepal [17]. Although the cause-specific mortality rate decreased by 27% between 2011 and 2017-18 in Bangladesh, pneumonia still causes approximately 24 000 deaths per year [9]. The majority of these deaths can be prevented by adopting the WHO recommended *prevent, protect and treat strategy* and by scaling up low-cost interventions [18]. However, there are substantial gaps in the coverage and quality of priority interventions outlined in WHO's *prevent, protect and treat strategy [*19,20*]*. In 2020, the median Global Action Plan for Pneumonia and Diarrhoea (GAPPD) pneumonia score was 81% in Bangladesh against the global target of 90% [21]. GAPPD scores are calculated as the average of 7 relevant indicators, which are as follows: pneumonia specific indicators such as exclusive breastfeeding, MCV1 coverage, DTP3 coverage, Hib3 coverage, PCV3 coverage, appropriate care seeking, antibiotic treatment. Although the coverage of key vaccines was reasonably high (98% for Hib3 and 97% for PCV), only 65% of children received exclusive breastfeeding [2]. Among children with a suspected episode of pneumonia, only 46% were taken to an appropriate health care provider, and 65% received antibiotics [2,21]. Our analysis found that the awareness of parents, care-seeking practice, and antibiotic coverage were even lower among the deceased children. The WHO recommended IMCI is one of the key strategies adopted by the Government of Bangladesh for managing pneumonia [22]. The success of IMCI services will greatly influence Bangladesh's progress towards achieving the GAPPD target of preventing all avoidable pneumonia-related deaths by 2025 [18]. Although ensuring the service availability and readiness of health facilities is critical to managing pneumonia through IMCI services effectively, there are substantial gaps, particularly in rural facilities [23]. Unfortunately, two-thirds of the designated facilities in Bangladesh do not have an ARI timer or watch with second timing [24]. Approximately half of the facilities do not have an appropriate weighing scale which is essential for prescribing proper dosages of medicines (including antibiotics for pneumonia management) [24,25]. There are substantial gaps regarding the availability of antibiotics such as amoxicillin, which is essential for managing pneumonia [24,25]. Hypoxaemia is common among children with pneumonia and is one of the strongest predictors of mortality due to pneumonia [26-28]. Less than one-tenth of the facilities in Bangladesh have pulse oximeters [24]. The availability of pulse oximeters was not optimal even in referral hospitals as less than one-third of the district hospitals and one-fifth of the sub-district hospitals have readiness in this regard [24]. Moreover, one-third of the district hospitals and one-fourth of the sub-district hospitals do not have a reliable oxygen source which is essential for managing severe pneumonia with hypoxaemia [24,29]. Unfortunately, these critical gaps in readiness related to pneumonia management did not change between 2014 and 2017, as reported by two national surveys [24,30]. This can also explain the apparent stalling in the overall child mortality rate, which was observed in the corresponding period [1,2]. In addition to ensuring the adequate readiness of equipment, drugs, and logistics essential for managing pneumonia, strategic investment should be made to ensure universal assessment of hypoxaemia with pulse oximetry and supportive care with oxygen therapy [31,32].

In our review, the cause-specific mortality rate due to birth asphyxia was 7.3 per thousand live birth, making it the second-largest killer among children under five years of age and the major contributor among all neonatal deaths. Around three-fourth of them died on the day of birth, emphasising the importance of delivering the intervention within a short window of opportunity. Prevention and management of birth asphyxia requires specialised skills and services, which are more conducive to be arranged and ensured among facility births. A systematic review reported facility birth as one of the strongest interventions to avert birth asphyxia related deaths [33]. Although facility births have been increasing substantially and consistently in Bangladesh since 2000, we did not observe any notable difference in the cause-specific mortality rate due to birth asphyxia since 2010 [9]. We found that more than half of the deaths due to birth asphyxia had a facility birth. The apparent gaps in facility readiness and quality of obstetric care prevailing in public and private health facilities in Bangladesh can potentially explain these contradictory trends. The latest national survey conducted in 2017 reported that less than 5% of the facilities offering normal delivery services in Bangladesh met the WHO recommended minimum readiness criteria. The condition remained unchanged from the 2014 estimate [24,30]. Other studies have reported major gaps in the provision and experience of care related to childbirth in both public and private health facilities in Bangladesh [34-36]. Moreover, around two-thirds of the facilities offering normal delivery services did not have a suction apparatus, and approximately half of the facilities did not have a bag-mask in the delivery corner, only one-third of providers received in-service training on newborn resuscitation which is essential for the management of birth asphyxia [24,37]. It is also important to note that around half of the babies who died due to birth asphyxia had a home birth. Since more than one-third of the total births happen at home, primarily with untrained birth attendants, particular strategies should be taken to promote facility birth with optimum quality of care.

Complications related to prematurity and low birth weight cause approximately 15 000 lives per year in Bangladesh, making them the third most significant contributor of deaths among children under five years of age and the second major contributor among all neonatal deaths. Kangaroo Mother Care (KMC) is an evidence-based, cost-effective and high-impact intervention that can significantly reduce the risk of death due to prematurity and low birth weight [38]. The Government of Bangladesh adopted the WHO-recommended KMC services as the primary approach for averting prematurity-related deaths through the Promise Renewed Declaration in 2013 [39,40]. However, there are substantial gaps between the policy and practice. According to the national guideline, any stable newborn with a birth weight of less than 2000g should receive KMC. Each year an estimated 573 000 babies are born prematurely, and 192 000 babies have low birth weight (less than 2000 g) in Bangladesh [41]. Only 193 facilities offer KMC services in Bangladesh, which reveals the prominent gap in care provision. In 2020, approximately 5700 babies received KMC services from these facilities, implying that the coverage was extremely poor at below 5% [42]. This is consistent with our findings, where none of the deceased children included in our analysis reported having received KMC. Also, the average duration of stay in KMC facilities was around three days, against the minimum recommendation of 10-14 days [42,43]. It highlights the substantial gap in quality, which deters achieving effective coverage with this life-saving intervention. We also found that around three-fourth of the deaths due to prematurity and low birth weight occurs on the date of birth. Since the rate of facility birth is increasing rapidly in Bangladesh, identifying prematurity and low birth weight at birth and introducing KMC immediately after birth can be a low hanging fruit to increase the coverage [2,44] efficiently. These gaps in provision, coverage and quality of KMC services are not unique to Bangladesh, as the majority of the low-and middle-income countries practicing KMC face similar barriers and challenges [44]. In addition to health systems bottlenecks, there are issues related to community awareness, acceptability, access and other demand-side barriers. The operational plan for the current health sector programme does not have any specific initiatives addressing these demand-side barriers [45]. It is important to adopt a multipronged approach including health systems strengthening, monitoring and mentoring as well as strong community engagement and participation to achieve high and effective coverage of KMC and avert preventable deaths.

Antenatal corticosteroids, given to women in preterm labour, is a safe, effective and low-cost intervention that can significantly reduce the deaths due to prematurity, primarily reducing the risk of respiratory distress syndrome. A Cochrane review and meta-analysis found that antenatal corticosteroid can reduce death by 31% and incidence of respiratory distress syndrome by 34% [46]. Another meta-analysis found that antenatal corticosteroids can have a more significant impact in low-resource settings without the provision of advanced intensive care [47]. The Government of Bangladesh decided to incorporate the use of antenatal corticosteroid in the national programme in 2013 [39]. However, the programme implementation plan did not identify specific actions and allocate resources for the capacity development of health care workers and ensuring the availability of essential logistics [22,45]. Hence, this promising intervention could not significantly impact prematurity-related mortality due to the extremely low coverage. Such gaps in the overall policy prioritisation, programme planning, implementation can explain the apparent increase in prematurity and low birth weight specific mortality rates that was observed between 2011 and 2017-18 [2,9].

We report that the cause-specific mortality rate due to serious infections (primarily sepsis) was 3.8 per thousand live birth, making it the fourth-largest killer among children under five years of age. Around 90% of these deaths happened during the neonatal period emphasising the importance of focusing on this age group with appropriate programme planning and targeted interventions. The cause-specific mortality rate reduced by almost two-thirds between 2011 and 2017-18. The primary reason for this decline could be the pervasive use of broad-spectrum antibiotics, especially by informal care providers in rural settings which are widely available, accessible, and affordable [48-53]. The National Newborn Health Programme also changed its policy and programme approach to improve accessibility and increase treatment coverage. Until 2015, the WHO recommended inpatient hospital care for managing possible severe bacterial infections with multi-drug multi-dose injectable antibiotics for 7-10 days [54]. Based on the evidence from the Simplified Antibiotic Therapy Trial (SATT) and African Neonatal Sepsis Trial (AFRINEST) studies, WHO changed its guideline in 2015 and recommended treating the clinically less severe possible bacterial severe infections through outpatient care with simplified antibiotic regimens where referral is not feasible [55-58]. The Government of Bangladesh adopted the WHO recommendation, changed the existing guidelines and took initiatives to scale up sepsis management nationally with the simplified antibiotic regimens [59]. This could also explain the decline in serious infection-related mortality in Bangladesh. Besides these achievements, there are programme implementation gaps in ensuring the availability of essential drugs and logistics, adhering to the clinical management guidelines by health care providers, completing the antibiotic course by the caregivers and quality of care received at the referral hospitals [59]. We also found that almost all of those who died due to serious infections received antibiotics and around one-third were born prematurely. It indicates that these cases were more complicated and required more specialised and advanced care at referral hospitals [29]. There are substantial gaps in the quality of inpatient care, including diagnostics, antibiotics and supportive care, in primary and secondary level referral hospitals in Bangladesh [60,61]. Unfortunately, the current national newborn and child health programme does not have any specific activity for strengthening and monitoring the provision and quality of inpatient care in referral facilities. Without strategic focus and substantial investment in inpatient care, it will be difficult to further reduce the serious infections specific mortality in Bangladesh.

Drowning is among the top five causes of death in Bangladesh. Around half of these deaths happen in the third year of life, making it the largest killer among children after passing their first year of life. Although the cause-specific mortality rate has declined slightly between 2011 and 2017-18, the national child health programme does not have any specific activity for promoting awareness and prevention in this regard [9,22]. The lack of adequate childcare while parents are working may be among the main reasons for these deaths. A large scale intervention trial conducted in seven rural sub-districts in Bangladesh found that crèches are effective for preventing childhood drowning as the incidence rate declined 87 per 100 000 population per year at baseline to 43 per 1000 population per year in the post-implementation period [62]. Although the evidence is encouraging, it will require substantial investments and a multisectoral approach to adopt, introduce and scale up this intervention, primarily in the districts with high drowning-related mortality burden. In addition, other interventions focusing on education and information, blocking access to water bodies by employing barriers and regulations, provision of supervision, and acquisition of survival skills can be contextualised and tested to add impetus to the drowning prevention efforts [63].

Complications related to birth defects and congenital anomalies are responsible for around 6% of all deaths under five years of age in Bangladesh, making it an emerging public health concern [2]. Although several referral hospitals have recently started birth defects screening and surveillance, most of these facilities are situated in urban areas, mainly clustered in bigger cities. The programme coverage and clinical support to managing the complications of birth defects and congenital anomalies are sub-optimum and require strategic focus with significant investments. Moreover, reducing the mortality burden of birth defects and congenital anomalies will substantially depend on the prevention strategy and effective delivery of interventions adopting a life course approach [64]. Bangladesh needs to develop and implement an integrated plan for the prevention and control of common birth defects involving public health programmes like maternal, newborn and child health, nutrition, immunisation, and other stakeholders.

Lastly, around 40% of all deaths under five years of age in Bangladesh happen in health facilities, in contrast to only 15% among adults. This has important implications for the public health programmes as it indicates the changing dynamic of care-seeking from formal health care providers and health facilities for fatal childhood illnesses. Proper investments should be made in improving the provision and quality of emergency and inpatient care for averting preventable deaths. Simultaneously, it is also important to note that more than half of the deaths still happen at home, and some after seeking care from formal health care providers. The context and challenges faced by the parents of these children can be very different from those who effectively sought care. It is important to understand the context and investigate the care-seeking challenges these communities face for developing a people-centred, participatory and responsive health system.

### Strength and limitations

The major causes of death presented in this paper have been analysed using the most recently published nationally representative Bangladesh Health and demographic Survey 2017-18 [2]. This survey includes samples from all administrative divisions of Bangladesh and has appropriate representation from urban and rural areas and across different populations' socio-demographic characteristics. Therefore, our results can be generalised to the overall population of Bangladesh. The data were collected using the validated WHO Standard Verbal Autopsy Tool, which was adapted to the country context before finalisation. The physicians received a month-long training on assigning the causes of death based on the ICD-10 codes. The authors of this paper were heavily involved in the training of the data collectors and physicians. They were also an integral part of the data quality monitoring team, which adds to the quality and authenticity of the data used in this analysis.

Although the survey used a nationally representative sample, the sample size was not enough for providing disaggregated estimates by different background characteristics with an acceptable level of precision. We acknowledge the limitation of the sample size in reporting the difference in cause-specific mortality rates by disaggregatingvariables with appropriate statistical significance. We also acknowledge the issues with recall error and, recall bias as the past information was collected from a close family member of the deceased person. Moreover, the tools had questions regarding clinical details, which could be intimidating and inappropriate for the respondents to recall and report accurately. We also acknowledge that the cause of deaths was assigned by physicians, which may have issues with misclassification bias due to the variations in subjective interpretations. However, the physicians were extensively trained and documented detailed justification for assigning the cause of deaths based on a structured checklist. These measures have contributed in minimising the misclassification bias. Lastly, we used data from the most recent national survey (2017-18), but the anchoring year was 2015.

## CONCLUSIONS

Accurate and reliable Information on the cause of childhood mortality is essential for developing policies and designing programmes targeting the major burden of disease. Around half of all deaths among children under five years of age happen in the neonatal period, emphasising the importance of prioritising neonatal health interventions and initiatives. Pneumonia with other serious infections, birth asphyxia and prematurity, and low birth weight are responsible for more than half of all deaths of children under five years of age. Strengthening of existing maternal, neonatal and child health programmes may be helpful in averting the majority of these preventable deaths. We recommend special measures to prevent and control emerging public health challenges like birth defects and congenital anomalies.

## Additional material


Online Supplementary Document

## References

[1] National Institute of Population Research and Training (NIPORT), Mitra and Associates, ICF International. Bangladesh Demographic and Health Survey 2014. Dhaka, Bangladesh and Calverton, Maryland, USA: 2016.

[2] National Institute of Population Research and Training (NIPORT), Mitra and Associates, ICF International. Bangladesh Demographic and Health Survey 2017-18. Dhaka, Bangladesh and Calverton, Maryland, USA: 2020.

[3] Mikkelsen L Phillips DE AbouZahr C Setel PW De Savigny D Lozano R A global assessment of civil registration and vital statistics systems: monitoring data quality and progress. Lancet. 2015;386:1395-406. 10.1016/S0140-6736(15)60171-4 25971218

[4] United Nations Children Fund. Childinfo: Monitoring the situation of children and women. Under-five Mortality (U5MR). 2009. Available: URL: http://www.childinfo.org/mortality_underfive.php. Accessed: April 4, 2021.

[5] Office of Registrar GeneralAvailable: http://180.211.213.83/brisreport/ReportPage.aspx?rpno=7. Accessed: October 30, 2020.

[6] World Health Organization. Revealing the toll of COVID-19: a technical package for rapid mortality surveillance and epidemic response. 2020.

[7] Amouzou A Kante A Macicame I Antonio A Gudo E Duce P National Sample Vital Registration System: A sustainable platform for COVID-19 and other infectious diseases surveillance in low and middle-income countries. J Glob Health. 2020;10:020368. 33110562 10.7189/jogh.10.020368 PMC7568202

[8] World Health Organization. Verbal autopsy standards: ascertaining and attributing cause of death: World Health Organization; 2007.

[9] National Institute of Population Research and Training (NIPORT), Mitra and Associates, ICF International. Bangladesh Demographic and Health Survey 2011. Dhaka, Bangladesh and Calverton, Maryland, USA: 2013.

[10] Jetté N Quan H Hemmelgarn B Drosler S Maass C Oec D-G The development, evolution, and modifications of ICD-10: challenges to the international comparability of morbidity data. Med Care. 2010;48:1105-10. 10.1097/MLR.0b013e3181ef9d3e 20978452

[11] STATASTATA. 2020. Available: https://www.stata.com/. Accessed: 1 December 2020.

[12] Bangladesh Bureaur of Statistics. Bangladesh Population and Housing Census 2011. 2011.

[13] Bangladesh Bureaur of Statistics. Sample Vital Registration System. 2015.

[14] Chowdhury AMR Bhuiya A Chowdhury ME Rasheed S Hussain Z Chen LC The Bangladesh paradox: exceptional health achievement despite economic poverty. Lancet. 2013;382:1734-45. 10.1016/S0140-6736(13)62148-0 24268002

[15] Ahsan KZ Streatfield PK Ijdi R-E-, Escudero GM, Khan AW, Reza M. Fifteen years of sector-wide approach (SWAp) in Bangladesh health sector: an assessment of progress. Health Policy Plan. 2016;31:612-23. 10.1093/heapol/czv108 26582744 PMC4857486

[16] United Nations. The Sustainable Development Goals Report 2016. 2016.

[17] McAllister DA Liu L Shi T Chu Y Reed C Burrows J Global, regional, and national estimates of pneumonia morbidity and mortality in children younger than 5 years between 2000 and 2015: a systematic analysis. Lancet Glob Health. 2019;7:e47-57. 10.1016/S2214-109X(18)30408-X 30497986 PMC6293057

[18] World Health Organization. UNICEF. End preventable deaths by 2025: The integrated Global Action Plan for Pneumonia and Diarrhoea (GAPPD). France: 2013.

[19] Johns Hopkins Bloomberg School of Public Health. Pneumonia & Diarrhea Progress Report 2018. 2018.

[20] Johns Hopkins Bloomberg School of Public Health. Pneumonia & Diarrhea Progress Report 2019. 2019.

[21] Johns Hopkins Bloomberg School of Public Health. Pneumonia & Diarrhea Progress Report 2020. 2020.

[22] Ministry of Health and Family Welfare (MOHFW)-Government of Bangladesh. 4th Health, Population and Nutrition Sector Programmme (4th HPNSP): Operational Plan for Maternal, Neonatal, Child, & Adolescent Health (MNC&AH) (January 2017-June 2022). Dhaka: Government of Bangladesh; 2017.

[23] World Health Organization. Integrated Management of Childhood Illness: Global Survey Report. 2017.

[24] National Institute of Population Research and Training (NIPORT), Associates for Community and Population Research (ACPR), ICF International. Bangladesh Health Facility Survey 2017. Dhaka, Bangladesh: 2019.

[25] World Health Organization. Integrated Management of Childhood Illness (IMCI): Chart Booklet: World Health Organization; 2014.

[26] Subhi R Adamson M Campbell H Weber M Smith K Duke T The prevalence of hypoxaemia among ill children in developing countries: a systematic review. Lancet Infect Dis. 2009;9:219-27. 10.1016/S1473-3099(09)70071-4 19324294

[27] Reed C Madhi SA Klugman KP Kuwanda L Ortiz JR Finelli L Development of the Respiratory Index of Severity in Children (RISC) score among young children with respiratory infections in South Africa. PLoS One. 2012;7:e27793. 10.1371/journal.pone.0027793 22238570 PMC3251620

[28] Lazzerini M Sonego M Pellegrin MC Hypoxaemia as a mortality risk factor in acute lower respiratory infections in children in low and middle-income countries: systematic review and meta-analysis. PLoS One. 2015;10:e0136166. 10.1371/journal.pone.0136166 26372640 PMC4570717

[29] World Health Organization. Pocket book of hospital care for children: guidelines for the management of common childhood illnesses: World Health Organization; 2013. 24006557

[30] National Institute of Population Research and Training (NIPORT), Associates for Community and Population Research (ACPR), ICF International. Bangladesh Health Facility Survey 2014. Dhaka, Bangladesh: 2016.

[31] Duke T Subhi R Peel D Frey B Pulse oximetry: technology to reduce child mortality in developing countries. Ann Trop Paediatr. 2009;29:165-75. 10.1179/027249309X12467994190011 19689857

[32] Graham H Tosif S Gray A Qazi S Campbell H Peel D Providing oxygen to children in hospitals: a realist review. Bull World Health Organ. 2017;95:288-302. 10.2471/BLT.16.186676 28479624 PMC5407252

[33] Igboanugo S Chen A Mielke JG Maternal risk factors for birth asphyxia in low-resource communities. A systematic review of the literature. J Obstet Gynaecol. 2020;40:1039-55. 10.1080/01443615.2019.1679737 31825270

[34] Billah SM Chowdhury MAK Khan ANS Karim F Hassan A Zaka N Quality of care during childbirth at public health facilities in Bangladesh: a cross-sectional study using WHO/UNICEF ‘Every Mother Every Newborn (EMEN)’standards. BMJ Open Qual. 2019;8:e000596. 10.1136/bmjoq-2018-000596 31523736 PMC6711449

[35] Perkins J Rahman AE Mhajabin S Siddique AB Mazumder T Haider MR Humanised childbirth: the status of emotional support of women in rural Bangladesh. Sex Reprod Health Matters. 2019;27:1610277. 10.1080/26410397.2019.1610277 31533580 PMC7887950

[36] Winter R Yourkavitch J Wang W Mallick L Assessment of health facility capacity to provide newborn care in Bangladesh, Haiti, Malawi, Senegal, and Tanzania. J Glob Health. 2017;7:020509. 10.7189/jogh.07.020509 29423186 PMC5804038

[37] World Health Organization. Guidelines on basic newborn resuscitation. 2012. 23700652

[38] Boundy EO Dastjerdi R Spiegelman D Fawzi WW Missmer SA Lieberman E Kangaroo mother care and neonatal outcomes: a meta-analysis. Pediatrics. 2016;137:e20152238. 10.1542/peds.2015-2238 26702029 PMC4702019

[39] Ministry of Health and Family Welfare, Government of the People's Republic of Bangladesh. Committing to Child Survival: A Promise Renewed Declaration 2013. Dhaka, Bangladesh: 2013.

[40] Unicef. Committing to child survival: a promise renewed. eSocialSciences, 2015.

[41] Blencowe H Krasevec J de Onis M Black RE An X Stevens GA National, regional, and worldwide estimates of low birthweight in 2015, with trends from 2000: a systematic analysis. Lancet Glob Health. 2019;7:e849-60. 10.1016/S2214-109X(18)30565-5 31103470 PMC6560046

[42] Directorate General of Health Services (DGHS). District Health Information System (DHIS2). 2020. Available: https://centraldhis.mohfw.gov.bd/dhismohfw/dhis-web-visualizer/. Accessed: 1 November 2020.

[43] World Health Organization. Kangaroo mother care: Practical guide, 2003. Geneva: Department of Reproductive Health and Research, WHO: 2003.

[44] Seidman G Unnikrishnan S Kenny E Myslinski S Cairns-Smith S Mulligan B Barriers and enablers of kangaroo mother care practice: a systematic review. PLoS One. 2015;10:e0125643. 10.1371/journal.pone.0125643 25993306 PMC4439040

[45] Ministry of Health and Family Welfare (MOHFW)-Government of Bangladesh. 4th Health, Population and Nutrition Sector Programmme (4th HPNSP): Operational Plan for Maternal, Child, Reproductive, & Adolescent Health (MCRAH) (January 2017-June 2022). Dhaka: Government of Bangladesh; 2017.

[46] Roberts D Brown J Medley N Dalziel SR Antenatal corticosteroids for accelerating fetal lung maturation for women at risk of preterm birth. Cochrane Database Syst Rev. 2017;CD004454. 10.1002/14651858.CD004454.pub3 28321847 PMC6464568

[47] Mwansa-Kambafwile J Cousens S Hansen T Lawn JE Antenatal steroids in preterm labour for the prevention of neonatal deaths due to complications of preterm birth. Int J Epidemiol. 2010;39:i122-33. 10.1093/ije/dyq029 20348115 PMC2845868

[48] Mahmood SS Iqbal M Hanifi SMA Wahed T Bhuiya A Are ‘Village Doctors’ in Bangladesh a curse or a blessing? BMC Int Health Hum Rights. 2010;10:18. 10.1186/1472-698X-10-18 20602805 PMC2910021

[49] Rahman MH Agarwal S Tuddenham S Peto H Iqbal M Bhuiya A What do they do? Interactions between village doctors and medical representatives in Chakaria, Bangladesh. Int Health. 2015;7:266-71. 10.1093/inthealth/ihu077 25406239

[50] Darmstadt GL Syed U Patel Z Kabir N Review of domiciliary newborn-care practices in Bangladesh. J Health Popul Nutr. 2006;24:380-93. 17591335 PMC3001142

[51] Chowdhury SK Billah SM Arifeen SE Hoque DME Care-seeking practices for sick neonates: Findings from cross-sectional survey in 14 rural sub-districts of Bangladesh. PLoS One. 2018;13:e0204902. 10.1371/journal.pone.0204902 30261083 PMC6160193

[52] Ahmed S Sobhan F Islam A Barkat e K. Neonatal morbidity and care-seeking behaviour in rural Bangladesh. J Trop Pediatr. 2001;47:98-105. 10.1093/tropej/47.2.98 11336143

[53] Shah R Mullany LC Darmstadt GL Talukder RR Rahman SM Mannan I Determinants and pattern of care seeking for preterm newborns in a rural Bangladeshi cohort. BMC Health Serv Res. 2014;14:417. 10.1186/1472-6963-14-417 25242278 PMC4261985

[54] World Health Organization. Pocket book of hospital care for children: guidelines for the management of common illnesses with limited resources. Geneva, Switzerland.: 2011. 24006557

[55] World Health Organization. Guideline: Managing possible serious bacterial infection in young infants when referral is not feasible. Geneva: World Health Organization, 2015. 26447263

[56] Baqui AH Saha SK Ahmed ANU Shahidullah M Quasem I Roth DE Safety and efficacy of alternative antibiotic regimens compared with 7 day injectable procaine benzylpenicillin and gentamicin for outpatient treatment of neonates and young infants with clinical signs of severe infection when referral is not possible: a randomised, open-label, equivalence trial. Lancet Glob Health. 2015;3:e279-87. 10.1016/S2214-109X(14)70347-X 25841891

[57] Tshefu A Lokangaka A Ngaima S Engmann C Esamai F Gisore P Simplified antibiotic regimens compared with injectable procaine benzylpenicillin plus gentamicin for treatment of neonates and young infants with clinical signs of possible serious bacterial infection when referral is not possible: a randomised, open-label, equivalence trial. Lancet. 2015;385:1767-76. 10.1016/S0140-6736(14)62284-4 25842221

[58] Tshefu A Lokangaka A Ngaima S Engmann C Esamai F Gisore P Oral amoxicillin compared with injectable procaine benzylpenicillin plus gentamicin for treatment of neonates and young infants with fast breathing when referral is not possible: a randomised, open-label, equivalence trial. Lancet. 2015;385:1758-66. 10.1016/S0140-6736(14)62285-6 25842223

[59] Rahman AE Herrera S Rubayet S Banik G Hasan R Ahsan Z Managing possible serious bacterial infection of young infants where referral is not possible: Lessons from the early implementation experience in Kushtia District learning laboratory, Bangladesh. PLoS One. 2020;15:e0232675. 10.1371/journal.pone.0232675 32392209 PMC7213695

[60] Rahman AE Iqbal A Hoque DE Moinuddin M Zaman SB Rahman QS-u Managing Neonatal and Early Childhood Syndromic Sepsis in Sub-District Hospitals in Resource Poor Settings: Improvement in Quality of Care through Introduction of a Package of Interventions in Rural Bangladesh. PLoS One. 2017;12:e0170267. 10.1371/journal.pone.0170267 28114415 PMC5256881

[61] Rahman AE Hossain AT Zaman SB Salim N Ashish K Day LT Antibiotic use for inpatient newborn care with suspected infection: EN-BIRTH multi-country validation study. BMC Pregnancy Childbirth. 2021;21:229. 10.1186/s12884-020-03424-7 33765948 PMC7995687

[62] Alonge O Bishai D Wadhwaniya S Agrawal P Rahman A Dewan Hoque EM Large-scale evaluation of interventions designed to reduce childhood Drownings in rural Bangladesh: a before and after cohort study. Inj Epidemiol. 2020;7:17. 10.1186/s40621-020-00245-2 32389128 PMC7212604

[63] Leavy JE Crawford G Leaversuch F Nimmo L McCausland K Jancey J A review of drowning prevention interventions for children and young people in high, low and middle income countries. J Community Health. 2016;41:424-41. 10.1007/s10900-015-0105-2 26499822

[64] World Health Organization. Prevention and control of birth defects in South-East Asia region: Strategic framework (2013-2017). WHO Regional Office for South-East Asia, 2013.

