# Supplementary material for: Child mortality in Bangladesh – why, when, where and how? A national survey-based analysis
Source: J Glob Health. 2021 Sep 11;11:04052. doi: 10.7189/jogh.11.04052 (PMC8442576; doi:10.7189/jogh.11.04052)
Supplement: Online Supplementary Document [file jogh-11-04052-s001.pdf]

Supplementary Tables and Figures:

Figure S1: Physician review process of assigning the cause of death.

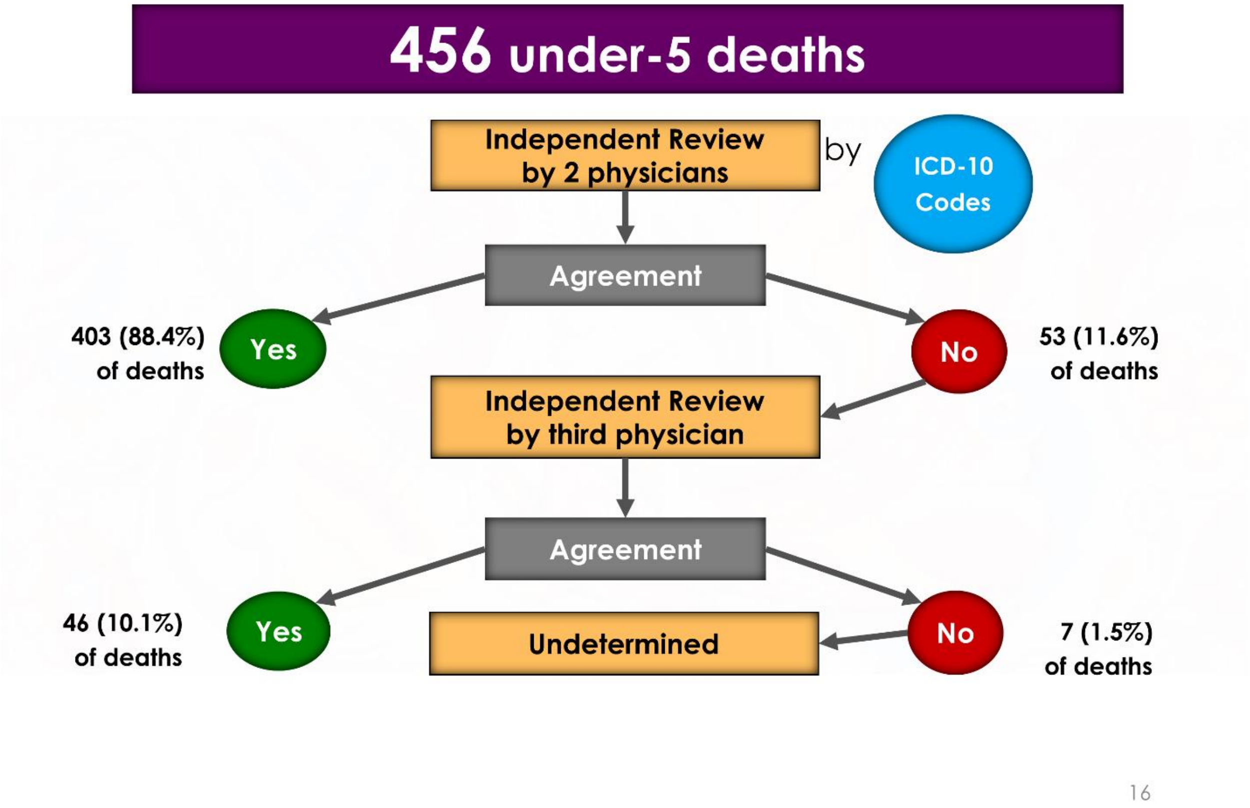

Figure S2: Comparison of cause specific mortality rates as the major cause of death between 2011 BDHS and 2017-18 BDHS.

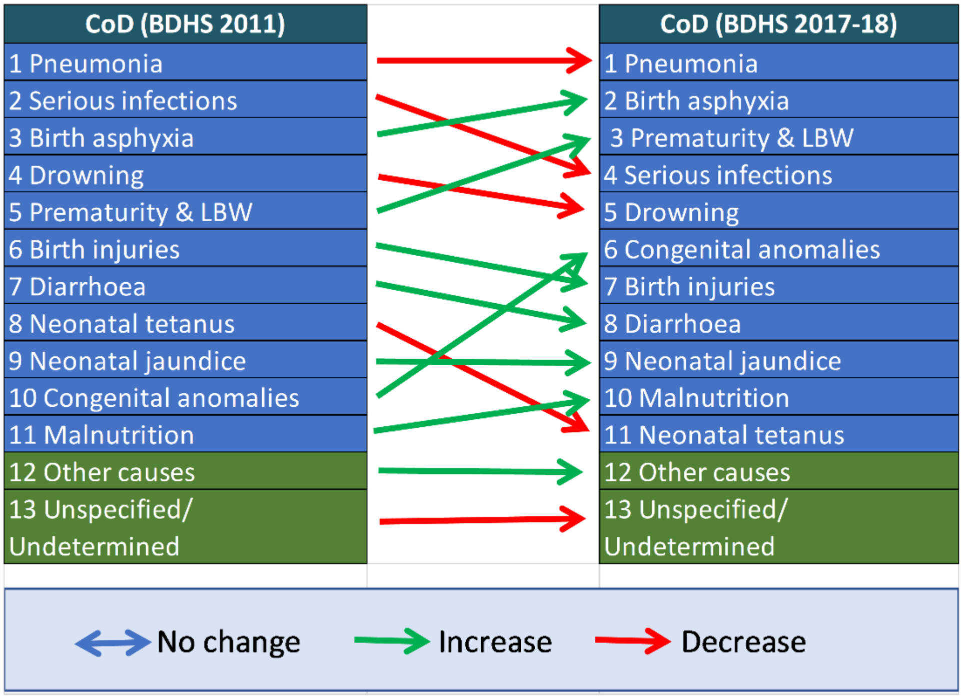

Table S1: Summery matrix of ICD-10 codes used to present the cause of death in categories

Broad Category	ICD-10 Code
1. Neonatal tetanus	A33
2. Congenital abnormality	Q00.0, Q02, Q03, Q03.9, Q24.9, Q42.3, Q43.9, Q74.9, Q75.9, Q76.9, Q79.5, Q89.9
3. Drowning	W70, W73
4. Birth asphyxia	P20.9, P21.9, P22.9, P24.0, P24.3, P24.9
5. Birth injury	P12.9, P13.1, P13.9, P15.9
6. Measles	
7. Diarrhoea	A05.9, A09.0
8. Pneumonia	J17.1, J18.9,
9. Meningitis	

10. Neonatal jaundice	P59.9, R17
11. Pre-term birth	P07.1, P07.3
12. Possible serious infection	A41.9, P36.9, P90
13. Malnutrition	E46,
14. Other causes	C26.0, C95.9, D56.9, D77, F89, G40.9, I51.9, K13.7, K76.9, N13.3, N17.9, N20.9, O75.9, P00.9, P01.2, P02.7, P51, P51.9, P70.2, P76.9, P91.7, R10.0, R50.9, R56, R56.0, R56.8, T63.0, W19, W87, Y09, Y65.2
15. Unspecified	P96.9, R99
16. Undetermined	Undetermined

Table S2: Projected number of deaths for each of the broad categories using the adjusted population size in 2015.

**Population 2011 from census 2011**	144043697			
**Growth rate from census 2011**	1.37%			
**CBR 2015 from SVRS 2015**	18.8			
**Projected population 2015**	152100991.6			
**Projected live birth in 2015**	2859499			
**Percent distributions for top eight causes**				
**Under 5 mortality**	45			
**Lower interval: Under 5 mortality**	40.1			
**Upper interval: Under 5 mortality**	49.5			
**Number of under 5 death**	128677			
**Lower limit: Number of under 5 death**	114666			
**Upper limit: Number of under 5 death**	141545			
**Percent distributions for top eight causes**	**Percent**	**Number of deaths**	**Lower limit**	**Upper limit**

Pneumonia	18.9	24268	21626	26695
Birth asphyxia	16.2	20882	18608	22970
Prematurity & LBW	11.6	14956	13327	16452
Serious infections	8.4	10723	9555	11795
Drowning	8.1	10441	9304	11485
Congenital anomalies	6.8	8748	7795	9623
Birth injuries	3.9	5079	4526	5587
Diarrhoea	3.1	3951	3520	4346

Table S3: Cause specific mortality by background characteristics, presented in deaths per thousand live births.

		Pneumonia	Birth asphyxia	Prematurity & LBW	Serious infections	Drowning	Congenital anomalies	Birth injuries	Diarrhoea
**Sex**	**Female**	8.3	7.0	5.2	4.8	3.7	2.2	1.7	1.7
	**Male**	8.0	7.0	4.8	2.7	3.3	3.5	1.7	1.0
**Birth Order**	**1**	8.1	9.6	5.6	4.0	2.0	4.5	3.5	0.5
	**2 or more**	11.1	7.4	6.4	4.5	5.8	2.7	0.8	2.5
**Mother Education**	**No education**	12.4	5.5	9.7	4.1	8.3	0.0	0.0	4.1
	**Primary Incomplete**	9.9	8.9	6.9	6.9	2.5	2.0	2.5	3.0
	**Primary Complete**	11.7	4.2	5.0	4.2	2.5	5.0	2.5	1.7
	**Secondary Incomplete**	7.5	7.8	4.6	2.5	4.1	3.7	0.9	0.7
	**Secondary Complete or More**	5.0	6.5	3.0	2.5	2.0	2.5	3.0	0.0
**Father Education**	**No education**	8.9	7.7	4.7	7.1	3.5	2.4	1.2	3.5
	**Primary Incomplete**	10.3	4.0	6.2	4.5	3.6	2.2	1.8	1.3
	**Primary Complete**	8.1	7.5	3.4	4.7	4.7	3.4	1.4	0.7
	**Secondary Incomplete**	7.1	7.9	6.6	2.1	3.7	3.3	2.9	1.2
	**Secondary Complete or More**	6.4	8.6	3.9	1.7	2.1	3.9	1.3	0.4
**Residence**	**Urban**	9.8	8.6	6.1	2.1	3.1	5.2	3.1	1.2

	**Rural**	7.6	6.5	4.7	4.4	3.7	2.0	1.1	1.4
**Wealth**	**Lowest**	10.6	7.2	5.1	4.7	5.1	1.7	1.3	3.4
	**Second**	6.1	7.1	6.6	4.7	2.8	1.4	0.9	0.9
	**Middle**	8.1	6.7	3.8	3.8	2.9	4.3	2.9	0.0
	**Fourth**	8.3	8.8	6.2	2.6	3.1	1.5	2.1	1.5
	**Highest**	8.1	6.0	3.8	2.2	3.2	6.5	1.6	0.5
**Total**		**8.5**	**7.3**	**5.2**	**3.8**	**3.7**	**3.1**	**1.8**	**1.4**
